# Occurrence and Importance of Yeasts in Indigenous Fermented Food and Beverages Produced in Sub-Saharan Africa

**DOI:** 10.3389/fmicb.2019.01789

**Published:** 2019-08-06

**Authors:** Pernille Greve Johansen, James Owusu-Kwarteng, Charles Parkouda, S. Wilfrid Padonou, Lene Jespersen

**Affiliations:** ^1^Department of Food Science, University of Copenhagen, Copenhagen, Denmark; ^2^Department of Food Science and Technology, University of Energy and Natural Resources, Sunyani, Ghana; ^3^Département Technologie Alimentaire, IRSAT/CNRST, Ouagadougou, Burkina Faso; ^4^ESTCTPA, Université Nationale d’Agriculture, Ketou, Benin

**Keywords:** yeast, indigenous fermented foods, sub-Sahara Africa, species and strain diversity, yeast successions, microbial interaction, sensorial and nutritional quality, health benefits

## Abstract

Indigenous fermented food and beverages represent a valuable cultural heritage in sub-Saharan Africa, having one of the richest selections of fermented food products in the world. In many of these indigenous spontaneously fermented food and beverages, yeasts are of significant importance. Several factors including raw materials, processing methods, hygienic conditions as well as the interactions between yeasts and other commensal microorganisms have been shown to influence yeast species diversity and successions. Both at species and strain levels, successions take place due to the continuous change in intrinsic and extrinsic growth factors. The selection pressure from the microbial stress factors leads to niche adaptation and both yeast species and strains with traits deviating from those generally acknowledged in current taxonomic keys, have been isolated from indigenous sub-Saharan African fermented food products. Yeasts are important for flavor development, impact shelf life, and nutritional value and do, in some cases, even provide host-beneficial effects. In order to sustain and upgrade these traditional fermented products, it is quite important to obtain detailed knowledge on the microorganisms involved in the fermentations, their growth requirements and interactions. While other publications have reported on the occurrence of prokaryotes in spontaneously fermented sub-Saharan food and beverages, the present review focuses on yeasts considering their current taxonomic position, relative occurrence and successions, interactions with other commensal microorganisms as well as beneficial effects and importance in human diet. Additionally, the risk of opportunistic yeasts is discussed.

## Yeasts as Contributors to Spontaneously Fermented Food and Beverages Produced in Sub-Saharan Africa

Fermentation is one of the oldest methodologies for food preservation and fermentation does therefore, globally play an important role in the processing of many indigenous food and beverages. Specifically, indigenous sub-Saharan Africa fermented food and beverages make up an import part of the daily diet in sub-Saharan African food culture, being of high nutritional value (Holzapfel, 2002). Fermented products are appreciated, not only due to the preservation and safety of these products but also because of their sensorial attributes (Holzapfel, 2002; Anukam and Reid, 2009). Moreover, several other beneficial effects of food fermentation have been reported including reduced loss of raw materials, reduced cooking time, prolonged shelf-life, enhanced bio-availability of micronutrients, and probiotic effects (Jespersen, 2003, 2005; Mufandaedza et al., 2006; Motlhanka et al., 2018). Most of these fermented products are produced in small-scale at small and medium-sized enterprises (SME) or at household level by spontaneous fermentation, sometimes including inoculation using back-slopping or repeated use of the same fermentation container (Holzapfel, 2002).

The most commonly used raw materials in indigenous sub-Saharan African fermented food and beverages are cereals, root tubers and legumes but other raw materials such as palm sap, milk, fruits, fish, and meat are also fermented (Jespersen, 2003; Ogunremi et al., 2017; Motlhanka et al., 2018). Indigenous fermented food and beverages are in general simple to process as they are typically based on a few ingredients and rely on simple processing methods (Marco et al., 2017). The nature of indigenous fermented food and beverages and the fact that they are easily accessible make these food systems ideal ecosystems for investigating the mechanisms of microbial community formation (Wolfe and Dutton, 2015). However, knowledge on the microbial ecology of indigenous sub-Saharan African fermented food and beverages lags behind.

The microorganisms involved in indigenous sub-Saharan African fermented food and beverages will predominantly originate from the raw materials and processing equipment as spontaneous fermentation or back-slopping is applied (Jespersen, 2003). The microbial consortium is complex, comprising a range of different microorganisms coexisting and interacting in many ways (Navarrete-Bolaños, 2012). Owing to their relatively high growth rate, lactic acid bacteria (LAB) will usually predominate the early stages of the fermentation, where after yeasts will increase and take part in the fermentation (Holzapfel, 2002).

The occurrence of yeasts in indigenous sub-Saharan African fermented food and beverage products has been studied for a range of end products. However, far fewer studies have examined the yeast dynamics during the fermentations. The yeast species dominating indigenous fermented food and beverages are those that are able to adapt to the changing intrinsic conditions caused by physicochemical changes, due to microbial activity (Navarrete-Bolaños, 2012). Species diversity is additionally influenced by different extrinsic factors related to the technological processing steps including length and temperature of fermentation, amount of water added, raw materials used, stirring, pasteurization as well as level of hygiene and sanitation (Jespersen, 2003; Achi and Ukwuru, 2015). Hence, a comprehensive understanding, linking intrinsic and extrinsic factors to microbial diversity and successions is of outmost importance for upgrading indigenous sub-Saharan African fermented food and beverages.

Several functional properties of yeasts have been reported for the processing of indigenous sub-Saharan African fermented food and beverages. These include fermentation of carbohydrates, flavor compound formation, stimulation of LAB, degradation of cyanogenic glycosides, production of tissue-degrading enzymes, binding and/or degradation of mycotoxins as well as probiotic properties (Jespersen, 2003, 2005; Omemu et al., 2007; Padonou et al., 2010; Achi and Ukwuru, 2015; Tamang et al., 2016). However, in most of the studied indigenous sub-Saharan African fermented food and beverages, the functional properties of the identified yeasts have not yet been extensively elucidated.

In general, information on yeasts in indigenous sub-Saharan African fermented food and beverages is very scattered, and though yeasts are considered very important for processing of local food and beverages, it is nearly impossible to get an overview on their identity, occurrence, impact and interactions. The aim of the present review is therefore to gather currently available scientific knowledge on yeasts involved in indigenous sub-Saharan African fermented food and beverages focusing on their current taxonomic position, species and strain diversity, microbial successions and interactions, as well as their impact on quality in terms of flavor, shelf life, safety and nutritional value. Reflections on health beneficial effects of yeasts, possible pathogenic traits as well as future technological improvements are likewise included. Although yeast species are also involved in the fermentation of cash crops such as coffee and cocoa (Masoud et al., 2004; Nielsen et al., 2005), these products are not included in the present survey.

## Species Diversity and Strain Distribution

### Taxonomic Methods for Identification of Yeasts

Methods used for identification of yeasts at species and strain levels can be classified roughly into two groups based on either phenotypic- or genotypic methodologies. Until recently, most publications dealing with yeasts in indigenous sub-Saharan African fermented food and beverages focused on identification based on phenotypic criteria. However, an enhanced availability of methods based on genotypic characterisation has changed the situation and current publications do to a greater extent base their identifications on genotypic methodologies. Below a brief overview on relevant methodologies for yeast identification is given.

Phenotypic methods include standard taxonomical tests, most recently described in the current taxonomic key edited by Kurtzman et al. (2011) as well as in a number of older publications such as the simplified identification method (SIM) described by Deak (2007). All of these methods are based on morphological and physiological characteristics (colony and cell morphology, growth conditions, assimilation and fermentation of carbohydrates as well as nitrogen compounds, osmotolerance, etc.). In the present survey, yeast species were reported for 43 indigenous sub-Saharan African fermented food and beverages, of which yeasts were identified by phenotypic approaches for 44% of the fermented products, as indicated in Tables 1–4. Identifications based only on phenotypic analyses can be doubtful as different species sometimes display very close morphological and physiological characteristics. Further, under certain conditions, strains might show different morphological and physiological traits, which deviate from the species description given in current taxonomic keys (Jespersen, 2003; Glover et al., 2005). While phenotypic characterisation provides useful information for a first grouping of isolates, it is nowadays recommended to include molecular identification analyses and vice versa (Kurtzman et al., 2015). Recently more advanced identification techniques based on phenotypic characteristics have been developed such as the matrix assisted laser desorption ionization-time of flight mass spectrometry (MALDI-TOF MS) (Pavlovic et al., 2014) and fourier transform infrared spectroscopy (FTIR) techniques (Wenning et al., 2002). However, these techniques have so far not been used for identification of yeasts from indigenous sub-Saharan African fermented food and beverages.

**TABLE 1:** Yeast species commonly identified in indigenous sub-Saharan African fermented solid foods.

Yeast species^*^	**Raw material**	**Product name**	**Associated bacteria and molds^∗∗^**	**Yeast count (log CFU/g)**	**Relative occurrence among yeasts^∗∗∗^**			**References**
						
					**≥50%**	**10–49%**	**<10%**	
*Candida glabrata*	Cereal	mawè	LAB	4.5–8.0		x	x	Greppi et al., 2013b; Hounhouigan et al., 1994^‡^; Houngbédji et al., 2018
	Tuber root	gari	LAB	4.0–6.0			x	Edward et al., 2012^‡^
	Tuber root	lafun	LAB, *Bacillus* spp.	4.2–5.8		x	x	Padonou et al., 2009
*Candida tropicalis*	Cereal	fura	–	–			x	Pedersen et al., 2012
	Cereal	gowé	LAB	4.9–6.2	x	x		Greppi et al., 2013a; Vieira-Dalodé et al., 2007
	Cereal	mawè	–	4.5–8.0		x	x	Greppi et al., 2013b
	Cereal	teff-injera	LAB	–		x		Desiye and Abegaz, 2013^‡^
	Tuber root	akyeke	*Bacillus* spp.	7.0–7.2	x			Obilie et al., 2003^‡^
	Tuber root	attiéké	LAB, *Bacillus* spp., mold	5.1	x			Coulin et al., 2006^‡^
	Tuber root	fufu^†^	–	5.2–8.0	x	x		Oyewole, 2001^‡^
	Tuber root	gari	LAB	4.0–6.0		x		Edward et al., 2012^‡^
	Tuber root	lafun	LAB, *Bacillus* spp.	4.2–5.8		x	x	Padonou et al., 2009
	Fish	adjuevan^†^	–	4.3–6.3		x	x	Clementine et al., 2012
*Debaryomyces hansenii*	Tuber root	gari	LAB	4.0–6.0			x	Edward et al., 2012^‡^
	Fish	adjuevan^†^	–	4.3–6.3	x	x		Clementine et al., 2012
	Fish	enam ne-setaakye	LAB	2.3–3.5	x			Asiedu and Sanni, 2002^‡^
*Kazachstania exigua*	Cereal	teff-injera	LAB	–			x	Desiye and Abegaz, 2013^‡^
	Palm	kocho	LAB	4.0–6.0			x	Birmeta et al., 2019
	Tuber root	attiéké	LAB, *Bacillus* spp., mold	4.9–8.3			x	Assanvo et al., 2006^‡^
*Kluyveromyces marxianus*	Cereal	fura	–	–		x		Pedersen et al., 2012
	Cereal	gowé	LAB	4.9–6.2	x	x	x	Greppi et al., 2013a; Vieira-Dalodé et al., 2007
	Cereal	mawè	LAB	4.5–8.0	x	x	x	Greppi et al., 2013a, 2013b; Hounhouigan et al., 1994^‡^; Houngbédji et al., 2018
	Tuber root	lafun	LAB, *Bacillus* spp.	4.2–5.8		x		Padonou et al., 2009
	Fish	adjuevan^†^	–	4.3–6.3		x		Clementine et al., 2012
*Pichia kudriavzevii*	Cereal	fura	–	–	x			Pedersen et al., 2012
	Cereal	gowé	LAB	4.9–6.2	x	x		Greppi et al., 2013a; Vieira-Dalodé et al., 2007
	Cereal	kenkey	LAB, mold	2.7–6.2	x		x	Halm et al., 1993^‡^; Jespersen et al., 1994^‡^
	Cereal	mawè	LAB	4.5–8.0	x	x		Greppi et al., 2013a, 2013b; Hounhouigan et al., 1994^‡^; Houngbédji et al., 2018
	Tuber root	attiéké	LAB, *Bacillus* spp., mold	4.9–8.3		x	x	Assanvo et al., 2006^‡^; Coulin et al., 2006^‡^
	Tuber root	fufu^†^	–	5.2–8.0	x	x		Oyewole, 2001^‡^
	Tuber root	gari	LAB	4.0–6.0		x		Edward et al., 2012^‡^
	Tuber root	lafun	LAB, *Bacillus* spp.	4.2–5.8		x	x	Padonou et al., 2009
*Saccharomyces cerevisiae*	Cereal	kenkey	LAB, mold	2.7–6.2	x	x		Halm et al., 1993^‡^; Jespersen et al., 1994
	Cereal	mawè	LAB	4.5–8.0	x	x	x	Greppi et al., 2013a, 2013b; Hounhouigan et al., 1994^‡^; Houngbédji et al., 2018
	Cereal	teff-injera	LAB	–		x		Desiye and Abegaz, 2013^‡^
	Tuber root	attiéké	LAB, *Bacillus* spp., mold	4.9–8.3		x	x	Assanvo et al., 2006^‡^; Coulin et al., 2006^‡^
	Tuber root	fufu^†^	–	5.2–8.0		x	x	Oyewole, 2001^‡^
	Tuber root	lafun	LAB, *Bacillus* spp.	4.2–5.8		x		Padonou et al., 2009
	Fish	adjuevan^†^	–	4.3–6.3		x	x	Clementine et al., 2012
*Wickerhamomyces anomalus*	Cereal	gowé	LAB	4.9–6.2		x		Vieira-Dalodé et al., 2007
	Cereal	mawè	LAB	4.5–8.0		x		Greppi et al., 2013b; Houngbédji et al., 2018
	Tuber root	fufu^†^	–	5.2–8.0			x	Oyewole, 2001^‡^

^*^Yeasts species reported in less than three indigenous sub-Saharan fermented solid foods are not included in the table. For all products more than one yeast species are involved. ^∗∗^Potential pathogenic bacteria excluded. ^∗∗∗^% of the total yeast population during fermentation and/or in the final product, as reported in at least one of the listed references. ^†^Relative occurrence based on description in the reference. ^‡^Identification based on phenotypic tests only. – not reported. LAB: lactic acid bacteria.

**TABLE 2:** Yeast species commonly identified in indigenous sub-Saharan African fermented non/low-alcoholic beverages.

Yeast species^*^	**Raw material**	**Product name**	**Associated bacteria and molds^∗∗^**	**Yeast count (log CFU/mL)**	**Relative occurrence^∗∗∗^**			**References**
						
					**≥50%**	**10–49%**	**<10%**	
*Candida tropicalis*	Cereal	ogi ^†^	–	2.9–7.9	x	x	x	Omemu et al., 2007^‡^
	Cereal	togwa	LAB	5.0–7.0		x	x	Mugula et al., 2003b^‡^
*Clavispora lusitaniae*	Cereal	obushera	LAB	3.5–7.6			x	Mukisa et al., 2012
	Cereal	ogi	–	2.9–7.9			x	Greppi et al., 2013a
*Pichia kudriavzevii*	Cereal	obushera	LAB	3.5–7.6			x	Mukisa et al., 2012
	Cereal	ogi	–	2.9–7.9	x	x		Greppi et al., 2013a; Omemu et al., 2007 ^‡^
	Cereal	togwa	LAB	5.0–7.0	x	x		Hellström et al., 2010; Mugula et al., 2003b^‡^
*Saccharomyces cerevisiae*	Cereal	obushera	LAB	3.5–7.6		x		Mukisa et al., 2012
	Cereal	ogi	–	2.9–7.9	x	x	x	Greppi et al., 2013a; Omemu et al., 2007^‡^
	Cereal	togwa	LAB	5.0–7.0		x		Hellström et al., 2010; Mugula et al., 2003b^‡^

^*^Yeasts species reported in less than two indigenous sub-Saharan fermented solid foods are not included in the table. For all products more than one yeast species are involved. ^∗∗^Potential pathogenic bacteria excluded. ^∗∗∗^% of the total yeast population during fermentation and/or in the final product, as reported in at least one of the listed references. ^†^Relative occurrence based on description in the reference. ^‡^Identification based on phenotypic tests only. – not reported. LAB: lactic acid bacteria.

**TABLE 3:** Yeast species commonly identified in indigenoussub-Saharan African fermented alcoholic beverages.

Yeast species^*^	**Raw material**	**Product name**	**Alcohol (%)**	**Associated bacteria and molds^∗∗^**	**Yeast count (log CFU/mL)**	**Relative occurrence^∗∗∗^**			**References**
							
						**≥50%**	**10–49%**	**<10%**	
*Candida tropicalis*	Cereal	kaffir^†^	–	LAB	8.0		x		van Der Walt, 1956^‡^
	Cereal	pito	2.1	–	–		x		Sefa-Dedeh et al., 1999^‡^
	Cereal	tchapalo	3.6–4.8	–	6.0–7.0		x	x	N’guessan et al., 2011
	Palm	bandji	0.3–2.7	LAB, AAB	4.8–7.3		x		Ouoba et al., 2012
	Palm	palm wine	–	–	–			x	Ezeronye and Okerentugba, 2001^‡^
	Fruit	mukumbi	–	–	7.6–7.7			x	Mpofu et al., 2008^‡^
*Hanseniaspora guilliermondii*	Cereal	tchoukoutou	–	–	5.0–6.5		x	x	Greppi et al., 2013b
	Palm	palm wine	3.7–4.4	–	7.2–7.4			x	Tra Bi et al., 2016
	Fruit	masau	–	LAB	1.2–9.3			x	Nyanga et al., 2007
	Fruit	pineapple wine	7.7	–	–	x			Dellacassa et al., 2017
*Hanseniaspora uvarum*	Cereal	kaffir^†^	–	LAB	8.0		x	x	van Der Walt, 1956^‡^
	Cereal	pito	2.1	-	-			x	Sefa-Dedeh et al., 1999^‡^
	Palm	bandji	0.3–2.7	LAB, AAB	4.8–7.3			x	Ouoba et al., 2012
	Palm	palm wine	3.2– > 7	LAB, AAB	7.5–8.2			x	Amoa-Awua et al., 2007^‡^
	Fruit	pineapple wine	7.7	–	–	x			Dellacassa et al., 2017
*Kluyveromyces marxianus*	Cereal	bili bili	–	–	–	x	x		Maoura et al., 2005
	Cereal	dolo	–	–	6.3–7.9			x	van der Aa Kühle et al., 2001
	Cereal	doro/chibuku	–	–	7.9–9.6			x	Misihairabgwi et al., 2015^‡^
	Cereal	kaffir^†^	–	LAB	8.0			x	van Der Walt, 1956^‡^
	Honey	tej	–	LAB	6.5–7.0		x		Bahiru et al., 2006^‡^
*Kodamea ohmeri*	Cereal	tchapalo	3.6–4.8	–	6.0–7.0			x	N’guessan et al., 2011
	Palm	bandji	0.3–2.7	LAB, AAB	4.8–7.3			x	Ouoba et al., 2012
	Palm	palm wine	3.4–3.7	LAB, AAB	7.5–8.2		x		Tra Bi et al., 2016
*Pichia kudriavzevii*	Cereal	burukutu^†^	4.5	LAB	8.2		x		Atter et al., 2014^‡^
	Cereal	doro/chibuku	–	–	7.9–9.6		x		Misihairabgwi et al., 2015^‡^
	Cereal	ikigage	2.2	LAB, mold	7.0		x		Lyumugabe et al., 2010^‡^
	Cereal	kaffir^†^	–	LAB	8.0		x		van Der Walt, 1956^‡^
	Cereal	tchapalo	3.6–4.8	–	6.0–7.0			x	N’guessan et al., 2011
	Cereal	tchoukoutou	3.2– > 7	–	5.0–6.5	x	x	x	Greppi et al., 2013a, b
	Palm	bandji	0.3–2.7	LAB, AAB	4.8–7.3	x	x		Ouoba et al., 2012
	Palm	palm wine	3.2– > 7	LAB, AAB	7.5–8.2			x	Amoa-Awua et al., 2007^‡^; Tra Bi et al., 2016
	Fruit	masau	–	LAB	1.2–9.3		x		Nyanga et al., 2007
*Saccharomyces cerevisiae*	Cereal	bili bili	–	–	–	x	x		Maoura et al., 2005
	Cereal	burukutu^†^	4.5	LAB	8.2	x			Atter et al., 2014^‡^
	Cereal	dolo	–	–	6.3–7.9	x			van der Aa Kühle et al., 2001
	Cereal	doro/chibuku	–	–	7.9–9.6	x			Misihairabgwi et al., 2015^‡^
	Cereal	ikigage	2.2	LAB, mold	7.0	x			Lyumugabe et al., 2010^‡^
	Cereal	kaffir^†^	–	LAB	8.0	x	x		van Der Walt, 1956^‡^
	Cereal	tchapalo	3.6–4.8	–	6.0–7.0	x			N’guessan et al., 2011
	Cereal	tchoukoutou	–	–	5.0–6.5	x	x	x	Greppi et al., 2013a, 2013b; Kayode et al., 2011^‡^
	Cereal	pito	2.1	–	–		x		Sefa-Dedeh et al., 1999^‡^
	Palm	bandji	0.3–2.7	LAB, AAB	4.8–7.3	x	x		Ouoba et al., 2012
	Palm	palm wine	3.2– > 7	LAB, AAB	7.5–8.2	x	x		Amoa-Awua et al., 2007^‡^; Ezeronye and Okerentugba, 2001^‡^; Stringini et al., 2009; Tra Bi et al., 2016
	Fruit	masau	–	LAB	1.2–9.3		x		Nyanga et al., 2007
	Fruit	mukumbi	–	–	7.6–7.7		x		Mpofu et al., 2008^‡^
	Honey	tej	–	LAB	6.5–7.0		x		Bahiru et al., 2006^‡^
*Schizosaccharomyces pombe*	Cereal	pito	2.1	–	–			x	Sefa-Dedeh et al., 1999^‡^
	Palm	bandji	0.3–2.7	LAB, AAB	4.8–7.3			x	Ouoba et al., 2012
	Palm	palm wine	–	–	–		x		Ezeronye and Okerentugba, 2001^‡^
*Torulaspora delbrueckii*	Cereal	bili bili	–	–	–			x	Maoura et al., 2005
	Cereal	pito	2.1	–	–			x	Sefa-Dedeh et al., 1999^‡^
	Cereal	tchoukoutou	–	LAB	8.9–9.1			x	Kayode et al., 2011^‡^
*Wickerhamomyces anomalus*	Cereal	kaffir^†^	–	LAB	8.0			x	van Der Walt, 1956^‡^
	Cereal	pito	2.1	–	–			x	Sefa-Dedeh et al., 1999^‡^
	Fruit	mukumbi	–	–	7.6–7.7			x	Mpofu et al., 2008^‡^
	Fruit	pineapple wine	7.7	–	–		x		Dellacassa et al., 2017

^*^Yeasts species reported in less than three indigenous sub-Saharan fermented solid foods are not included in the table. For all products more than one yeast species are involved. ^∗∗^Potential pathogenic bacteria excluded. ^∗∗∗^% of the total yeast population during fermentation and/or in the final product, as reported in at least one of the listed references. ^†^Relative occurrence based on description in the reference. ^‡^Identification based on phenotypic tests only. – not reported. LAB: lactic acid bacteria. AAB: acetic acid bacteria.

**TABLE 4:** Yeast species commonly identified in indigenous sub-Saharan African fermented dairy products.

Yeast species^*^	**Product name**	**Associated bacteria and molds^∗∗^**	**Yeast count (log CFU/mL)**	**Relative occurrence^∗∗∗^**			**References**
					
				**≥50%**	**10–49%**	**<10%**	
*Candida parapsilosis*	lait caillé	LAB	3.6–6.4	x	x	x	Bayili et al., 2019
	nunu	LAB	2.8–6.0		x	x	Akabanda et al., 2013
	sethemi^†^	LAB	3.8–6.2			x	Kebede et al., 2007
*Candida rugosa*	amasi	-	2.0–8.1			x	Gadaga et al., 2000^‡^
	nunu	LAB	2.8–6.0		x	x	Akabanda et al., 2013
	sethemi^†^	LAB	3.8–6.2			x	Kebede et al., 2007^‡^
	suusac	-	2.0–5.4			x	Njage et al., 2011
*Candida tropicalis*	amasi	-	2.0–8.1			x	Gadaga et al., 2000^‡^
	nunu	LAB	2.8–6.0		x	x	Akabanda et al., 2013
	sethemi^†^	LAB	3.8–6.2			x	Kebede et al., 2007
*Clavispora lusitaniae*	amasi	-	2.0–8.1		x		Gadaga et al., 2000^‡^
	sethemi^†^	LAB	3.8–6.2			x	Kebede et al., 2007
	suusac	–	2.0–5.4			x	Njage et al., 2011
*Debaryomyces hansenii*	amabere amaruranu	LAB	4.7–6.1		x		Nyambane et al., 2014^‡^
	kefir	LAB, AAB	5.2–8.6			x	Loretan et al., 2003^‡^
	sethemi^†^	LAB	3.8–6.2		x		Kebede et al., 2007
	suusac	–	2.0–5.4		x	x	Njage et al., 2011
*Kluyveromyces marxianus*	amasi	–	2.0–8.1			x	Gadaga et al., 2000^‡^
	gariss	LAB	6.1–7.8	x	x		Abdelgadir et al., 2008
	kefir	LAB, AAB	5.2–8.6	x	x	x	Loretan et al., 2003^‡^; Witthuhn et al., 2004^‡^,
	mashita	LAB	–		x		Ongol and Asano, 2009
	mursik	LAB	–		x		Nieminen et al., 2012
	nunu	LAB	2.8–6.0		x		Akabanda et al., 2010^‡^
	rob	LAB	6.3–7.6		x		Abdelgadir et al., 2001
	sethemi^†^	LAB	3.8–6.2		x	x	Kebede et al., 2007
*Papiliotrema laurentii*	amasi	–	2.0–8.1			x	Gadaga et al., 2000^‡^
	sethemi^†^	LAB	3.8–6.2			x	Kebede et al., 2007
	suusac	-	2.0–5.4			x	Njage et al., 2011
*Pichia kudriavzevii*	amasi	–	2.0–8.1			x	Gadaga et al., 2000^‡^
	gariss	LAB	6.1–7.8	x	x		Abdelgadir et al., 2001
	mashita	LAB	–	x			Ongol and Asano, 2009
	mursik	LAB	–	x			Nieminen et al., 2012
	nunu	LAB	2.8–6.0	x	x		Akabanda et al., 2013
	suusac	–	2.0–5.4	x		x	Lore et al., 2005^‡^; Njage et al., 2011
*Rhodotorula mucilaginosa*	amasi	–	2.0–8.1			x	Gadaga et al., 2000^‡^
	sethemi^†^	LAB	3.8–6.2			x	Kebede et al., 2007
	suusac	–	2.0–5.4			x	Lore et al., 2005^‡^; Njage et al., 2011
*Saccharomyces cerevisiae*	amabere amaruranu	LAB	4.7–6.1		x		Nyambane et al., 2014^‡^
	amasi	–	2.0–8.1		x		Gadaga et al., 2000^‡^
	kefir	LAB	5.2–8.6		x		Loretan et al., 2003^‡^; Witthuhn et al., 2004^‡^,
	lait caillé	LAB	3.6–6.4	x	x	x	Bayili et al., 2019
	mashita	LAB	–		x		Ongol and Asano, 2009
	nunu	LAB	2.8–6.0	x	x		Akabanda et al., 2010^‡^, 2013
	nyarmie	LAB	6.9–7.5		x		Obodai and Dodd, 2006^‡^
	rob	LAB	6.3–7.6	x			Abdelgadir et al., 2001
	sethemi^†^	LAB	3.8–6.2		x		Kebede et al., 2007
	suusac	–	2.0–5.4	x			Njage et al., 2011
*Torulaspora delbrueckii*	amasi	–	2.0–8.1			x	Gadaga et al., 2000^‡^
	kefir	LAB, AAB	5.2–8.6			x	Loretan et al., 2003^‡^; Witthuhn et al., 2004^‡^
	mursik	LAB	–			x	Nieminen et al., 2012
	sethemi^†^	LAB	3.8–6.2			x	Kebede et al., 2007
*Yarrowia lipolytica*	amasi	–	2.0–8.1			x	Gadaga et al., 2000^‡^
	kefir	LAB, AAB	5.2–8.6	x	x		Witthuhn et al., 2004^‡^
	nunu	LAB	2.8–6.0			x	Akabanda et al., 2010^‡^
	sethemi^†^	LAB	3.8–6.2			x	Kebede et al., 2007

^*^Yeasts species reported in less than three indigenous sub-Saharan fermented solid foods are not included in the table. For all products more than one yeast species are involved. ^∗∗^Potential pathogenic bacteria excluded. ^∗∗∗^% of the total yeast population during fermentation and/or in the final product, as reported in at least one of the listed references. ^†^Relative occurrence based on description in the reference. ^‡^Identification based on phenotypic tests only. – not reported. LAB: lactic acid bacteria. AAB: acetic acid bacteria.

Genotypic identification to species level is for yeasts to a great extent based on sequencing of the D1/D2 region of the 26S rRNA gene and to some extent based on sequencing of the internal transcribed spacer (ITS) regions (divided into ITS1 and ITS2 by the conserved 5.8S rRNA gene) (Jespersen, 2003; Daniel et al., 2014; Kurtzman et al., 2015). Genotypic identification to strain level is based on other specific molecular techniques including chromosome length polymorphism (CLP) analysis by pulse field gel electrophoresis (PFGE) (van der Aa Kühle et al., 2001; Glover et al., 2005; Maoura et al., 2005; Ezeronye and Legras, 2009), restriction fragment length polymorphism (RFLP) of the ITS regions using restriction enzymes such as *Hae*III, *Eco*RI, *Hind*III, *Hinf*I, *Dra*I, *Hpa*II, *Scr*FI, *Taq*I, etc. (Hayford and Jakobsen, 1999; van der Aa Kühle et al., 2001; Naumova et al., 2003; Glover et al., 2005; Maoura et al., 2005; Ezeronye and Legras, 2009; N’guessan et al., 2011), sequencing analyses of the *ACT1* and/or *COX2* genes (Glover et al., 2005; Ezeronye and Legras, 2009; N’guessan et al., 2011), microsatellite loci analyses (Naumova et al., 2003; Ezeronye and Legras, 2009; Stringini et al., 2009; Tapsoba et al., 2015; Tra Bi et al., 2016) as well as detection of specific loci such as the *MAL* loci distribution in *Saccharomyces cerevisiae* strains (van der Aa Kühle et al., 2001).

### Yeast Species Diversity and Distribution

**FIGURE 1:**
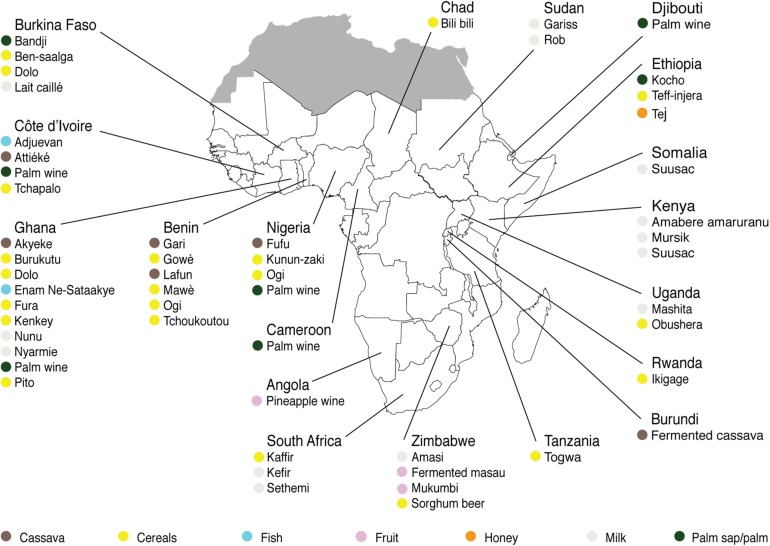
Origin and raw materials used for production of indigenous sub-Saharan African fermented food and beverages.

For each indigenous sub-Saharan African fermented food and beverage included in this review, the country of origin and raw material used can be found in Figure 1. Based on the current available literature, the fermented food and beverages have been classified into solid foods, non/low-alcoholic beverages, alcoholic beverages and fermented dairy products, in this review, even though it is acknowledged that other ways of classifying food fermentations exist (Steinkraus, 1997; Soro-Yao et al., 2014). The most frequently occurring yeast species are shown for the four groups in Tables 1–4, respectively. In order to use just one name for each yeast species, yeast species mentioned in Tables 1–4 and in the remaining parts of this review are only reported with their current taxonomic name, but mentioning the first time frequently reported former names, and for teleomorph (perfect) yeasts their anamorph (imperfect) name, if relevant. Differentiation between *Hanseniaspora guilliermondii* (anamorph *Kloeckera apis*) and *Hanseniaspora opuntiae* is not done, since these species cannot be distinguished by physiological criteria, and only the ITS region provide accurate identification as the D1/D2 region between the two species is highly conserved (Cadez et al., 2003).

From the 43 indigenous sub-Saharan African fermented food and beverages investigated, a total of 98 different yeast species have been identified. *S. cerevisiae* was reported in 77% of the fermented products, being the predominant yeast species isolated, followed by *Pichia kudriavzevii* (f. *Issatchenkia orientalis*; anamorph *Candida krusei*) reported in 60%, *Candida tropicalis* in 47% and *Kluyveromyces marxianus* (anamorph *Candida kefyr*) in 44% of the fermented products.

Not surprisingly, *S. cerevisiae* is the most frequently occurring yeast across all indigenous sub-Saharan African fermented food and beverages, and moreover, reported as the dominant yeast species in many of the foods it is being isolated from. Hence, *S. cerevisiae*, plays a dominant role in fermentation of solid foods such as mawè (Greppi et al., 2013b) and kenkey (Halm et al., 1993; Table 1), as well as in the non/low-alcoholic beverage ogi (Omemu et al., 2007; Table 2). Expectedly, *S. cerevisiae* is the most frequently occurring yeast in alcoholic beverages and has been reported to dominate the fermentation in 93% of the indigenous sub-Saharan African alcoholic beverages. The majority of these alcoholic beverages are based on cereals (Table 3). However, *S. cerevisiae* also dominates in other alcoholic beverages such as palm wine based on sap (Amoa-Awua et al., 2007; Ezeronye and Legras, 2009; Stringini et al., 2009). *S. cerevisiae* has additionally been reported to dominate the fermentation of the fermented dairy products nunu (Akabanda et al., 2010, 2013), rob (Abdelgadir et al., 2001), and suusac (Njage et al., 2011; Table 4). In lower abundancies, *S. cerevisiae* has been reported in other indigenous sub-Saharan African fermented products such as the solid foods adjuevan (Clementine et al., 2012), fufu (Oyewole, 2001), and lafun (Padonou et al., 2009), and the non/low-alcoholic beverages obushera (Mukisa et al., 2012) and togwa (Mugula et al., 2003b; Hellström et al., 2010), as well as the fermented dairy products amabere amaruranu (Nyambane et al., 2014), amasi (Gadaga et al., 2000), kefir (Loretan et al., 2003; Witthuhn et al., 2004), mashita (Ongol and Asano, 2009), nyarmie (Obodai and Dodd, 2006) and sethemi (Kebede et al., 2007; Tables 1, 2, 4).

Interestingly, *P. kudriavzevii* quite often plays a significant role in the fermentation of indigenous sub-Saharan African food and beverages. Previous studies demonstrated that *P. kudriavzevii* dominates the fermentations of several solid fermented products such as fufu (Oyewole, 2001), fura (Pedersen et al., 2012), gowé (Greppi et al., 2013a), kenkey (Halm et al., 1993), and mawè (Greppi et al., 2013b; Houngbédji et al., 2018; Table 1). Likewise, *P. kudriavzevii* has been shown to dominate in the fermentation of non/low-alcoholic beverages such as ogi (Omemu et al., 2007) and togwa (Mugula et al., 2003b; Hellström et al., 2010; Table 2). In alcoholic cereal-based beverages *P. kudriavzevii* is present, though not being the dominant species, except in bandji (Ouoba et al., 2012) and tchoukoutou (Greppi et al., 2013a,b; Table 3). Further, *P. kudriavzevii* has been reported to dominate several fermented dairy products such as gariss (Abdelgadir et al., 2008), mashita (Ongol and Asano, 2009), mursik (Nieminen et al., 2012), nunu (Akabanda et al., 2013), and suusac (Lore et al., 2005; Table 4).

Likewise, *C. tropicalis* and *K. marxianu*s are often found in indigenous sub-Saharan African fermented food and beverages. *C. tropicalis* is most frequently isolated from solid foods, being identified in 71% of the solid foods. *C. tropicalis* has been reported to dominate the fermentation of the solid foods akyeke (Obilie et al., 2003), attiéké (Coulin et al., 2006), fufu (Oyewole, 2001), and gowé (Greppi et al., 2013a). However, in most solid foods *C. tropicalis* does not dominate the fermentations (Table 1). *C. tropicalis* is not frequently associated with fermented dairy products and does only occur in low abundance in amasi (Gadaga et al., 2000), nunu (Gadaga et al., 2000; Akabanda et al., 2013), and sethemi (Kebede et al., 2007; Table 4). *K. marxianus* has been found to be dominant in the solid foods adjuevan (Clementine et al., 2012), gowé, (Greppi et al., 2013a), and mawè (Houngbédji et al., 2018; Table 1), as well as in the alcoholic beverage bili bili (Maoura et al., 2005; Table 3). Likewise, *K. marxianus* does mostly occur in fermented dairy products, being identified in 73% fermented dairy products, and is reported to dominate in the fermentation of the dairy products gariss (Abdelgadir et al., 2008), and kefir (Loretan et al., 2003; Witthuhn et al., 2004; Table 4). A novel yeast species, i.e., *Hanseniaspora jakobsenii* was isolated from bandji, a traditional fermented palm wine in Burkina Faso (Ouoba et al., 2015). The huge diversity of yeast species associated with indigenous sub-Saharan African fermented food and beverages underlines the high potential within the microbial heritage of these fermented food and beverages.

### Successions of Yeasts During Fermentations

During spontaneous fermentation of food and beverages, a complex yeast population is often seen. The diversity and relative composition of the yeast population will usually vary along the fermentation and successions will take place (Fleet, 2007). The succession of yeast species depends on various intrinsic and extrinsic factors related to the food matrix including any microbial interactions. While certain species occur throughout the fermentation, other species might appear or disappear as the fermentation progresses (Navarrete-Bolaños, 2012). Generally, in the early stages of the spontaneous fermentations a large number of yeast species are present. These are often characterized by low tolerance to microbial stress factors as ethanol and organic acids. As the fermentation progresses and the acid and alcohol contents increase, the more stress tolerant yeast species take over resulting in far fewer species completing the fermentation (Ganucci et al., 2018). In order to understand fully the microbial successions occurring during spontaneous fermentations, it is important to get a comprehensive overview on the entire microbiota involved in the process.

In general, two different approaches can be applied for analyzing the microbiota from complex foods. Presumptive yeasts can either be quantified by a culture-dependent approach, initially isolating the yeasts on appropriate media followed by species identification. Alternatively, the yeast community can be quantified by culture-independent methodologies such as DGGE (Ongol and Asano, 2009; Stringini et al., 2009; Clementine et al., 2012; Mukisa et al., 2012; Greppi et al., 2013b) or amplicon-based high-through put sequencing (Cocolin and Ercolini, 2015). Although accurate, culture-dependent methodologies are limited by the selective media used and will not be able to identify viable but non-culturable yeasts. On the other hand, culture-independent methods are unable to distinguish between living and dead microorganisms when quantifications are based on DNA (Cocolin and Ercolini, 2015). Combining both culture-dependent and -independent methodologies have been applied to study yeast diversity in mawè and tchoukoutou (Greppi et al., 2013b) as well as in palm wine (Stringini et al., 2009). Some few examples of yeasts successions in indigenous sub-Saharan African fermented food and beverages are given below.

Among the solid foods, microbial successions have been reported in the cereal-based mawè, in which *P. kudriavzevii* and *K. marxianus* have been shown to be present throughout the fermentation, i.e., 72 h (Greppi et al., 2013b; Houngbédji et al., 2018). In the study by Greppi et al. (2013b), *S. cerevisiae* was reported after 24 h and remained present until the end of the fermentation, while *C. tropicalis*, *Wickerhamomyces anomalus* (f. *Hansenula anomala*, *Pichia anomala*; anamorph *Candida pelliculosa*), *Millerozyma farinosa* (f. *Pichia farinosa*) and *Rhodotorula mucilaginosa* (f. *Rhodotorula rubra*) were reported only within 24 h of fermentation, though in low abundance. The opportunistic pathogenic yeast *Candida glabrata* was occasionally reported in high numbers during the initial stage of fermentation, i.e., 0–48 h, but disappeared at the end of the fermentation (Greppi et al., 2013b). In the solid food fufu based on cassava, *P. kudriavzevii*, *C. tropicalis*, *Saturnispora saitoi* (f. *Pichia saitoi*), *S. cerevisiae*, *W. anomalus* and *Zygosaccharomyces bailii* were isolated at the initial stage of the fermentation, i.e., 0–12 h (Oyewole, 2001). *W. anomalus*, *S. saitoi*, and *S. cerevisiae* disappeared within the first 48 h of fermentation, while *P. kudriavzevii*, *C. tropicalis*, and *Z. bailii* dominated the fermentation until the end, i.e., 96 h (Oyewole, 2001). In the solid food based on fermentation of enset palm into kocho, *Galactomyces geotrichum* (anamorph *Geotrichum candidum*), *Pichia exigua* and *Pichia fermentans* were reported as the most frequently isolated yeast species in fresh fermented kocho after 2–5 days of fermentation (Birmeta et al., 2019). As the fermentation progressed, *G. geotrichum* was still dominating after 3–4 months together with *Candida cabralensis* and *Candida ethanolica*. At the end of the fermentation (7–12 months) the dominant yeast species was *Saturnispora silvae* followed by *Cyberlindnera jadinii* (f. *Pichia fabianii*; anamorph *Candida fabianii*), while *G. geotrichum* only comprised a minor part of the yeasts (Birmeta et al., 2019).

Among the non/low-alcoholic beverages, microbial successions have been reported in the cereal-based ogi (Omemu et al., 2007). *S. cerevisiae* and *Rhodotorula graminis* dominated the steeping step. In the beginning of the fermentation, *S. cerevisiae* dominated, while the later part of the fermentation was dominated by *P. kudriavzevii*, *C. tropicalis*, *Dipodascus fermentans* (anamorph *Geotrichum fermentans*), and *G. geotrichum* (Omemu et al., 2007).

Among the alcoholic beverages, reports on microbial successions include the cereal-based tchoukoutou (Greppi et al., 2013b). *Clavispora lusitaniae* (anamorph *Candida lusitaniae*) followed by *P. kudriavzevii* was dominant for the first 4 h, while *S. cerevisiae* was found to dominate the fermentation from 4 h until the end of the fermentation, i.e., 12 h (Greppi et al., 2013b). According to Stringini et al. (2009), *Saccharomycodes ludwigii* was dominant at the beginning of palm wine fermentation. After 24 h *S. cerevisiae* increased in number while *S. ludwigii* decreased and could not be detected after 5 days of fermentation. Other yeast species as *Z. bailii*, *Hanseniaspora uvarum*, *Candida parapsilosis*, and *Meyerozyma caribbica* (anamorph *Candida fermentati*) were only reported during the first 3 days of fermentation, while *S. cerevisiae* was the only yeast species present from day 3–5 (Stringini et al., 2009).

Among the fermented dairy products, yeast successions was reported for nunu (Akabanda et al., 2013). Different successions occurred at the three production sites investigated. At one production site, only *P. kudriavzevii* and *S. cerevisiae* were reported in the fermentation. At the other two production sites, *C. parapsilosis*, *Candida rugosa*, and *C. tropicalis* were reported sporadically during the fermentation, with *G. geotrichum* making up a substantial part of the yeasts in the later stages of the fermentation at one of these sites (Akabanda et al., 2013). In the fermented dairy product lait caillé, *C. parapsilosis* was dominating the first 18 h of fermentation, while from 35 h until the end of the fermentation *S. cerevisiae* was dominating (Bayili et al., 2019).

### Strain Diversity of Yeasts

Diversity is not only observed at species level, also at strain level significant diversity exists. The stressful microbial environments in fermented food and beverages result in a high selection pressure, which can lead to development of new strains better adapted to the fermentation process. As a consequence, species occurring in spontaneously fermented food and beverages might, over time, differentiate into populations of strains of the same species (Suzzi, 2011).

Several investigations have been carried out to understand the diversity of *S. cerevisiae* strains in indigenous sub-Saharan African fermented food and beverages. Especially in sub-Saharan African beers, strain diversities have been investigated. *S. cerevisiae* strains with atypical phenotypic and genotypic traits have been isolated from West African sorghum beer (van der Aa Kühle et al., 2001). Among the 40 isolates, only 78% were able to ferment sucrose and none were able to ferment raffinose or trehalose. Further, 55% of the isolates had assimilation patterns quite atypical of *S.* cerevisiae, where several of the strains were unable to assimilate raffinose and some trehalose (van der Aa Kühle et al., 2001). Significant variations in *MAL* loci composition between *S. cerevisiae* strains from West African sorghum beer and European brewing strains were observed (van der Aa Kühle et al., 2001; Jespersen, 2003). For all *S. cerevisiae* isolates from sorghum beer, *MAL11* and *MAL31* were identified as the only recognized *MAL* loci, while the isolates lacked several of the *MAL* loci usually present in brewer’s and baker’s yeasts. Additionally, 40% of the isolates had an undescribed *MAL* locus of approximately 950 kb, which seemed to be specifically linked to this group of isolates. Similarly, based on phenotypic characteristics, sequencing of the ITS1 region of the 26S rRNA gene and Southern hybridization analysis, Naumova et al. (2003) reported that *S. cerevisiae* strains isolated from sorghum beer in Ghana and Burkina Faso represent a divergent population as compared to the European type strain CBS 1171. Further, only *SUC2* and *RTM* genes were detected in the sorghum beer strains, which is contrary to the European top and bottom fermenting brewing strains exhibiting multiple teleomeric *SUC* and *RTM* genes (Naumova et al., 2003). By whole genome sequencing of *S. cerevisiae* strains from around the world, distinct lineages correlating with geography, environmental niche and degree of human association have been reported in which, *S. cerevisiae* strains from African beers and palm wines were found to form a separate cluster (Gonçalves et al., 2016; Peter et al., 2018). Among the isolates, polyploidy (3–5*n*) was especially enriched in specific subpopulations, including the African beer and palm clades, which according to the authors strongly suggests that ploidy level is affected by human-related environments (Peter et al., 2018). Only observed among the West African wine strains, a specific type of inactivation of the *PAD1* gene, involved in decarboxylation of ferulic acid to the phenolic off-flavor compound 4-vinylguaiacol, was reported (Gonçalves et al., 2016). Through inter-delta and microsatellite analyses, *S. cerevisiae* strains isolated from Nigerian palm wine were reported to form an autochthonous population genetically distant from strains isolated from Ghanaian sorghum beers (Ezeronye and Legras, 2009). In a study using microsatellite analysis to compare *S. cerevisiae* strains isolated from palm wines in Burkina Faso, Djibouti, Nigeria, and Côte d’Ivoire, *S. cerevisiae* strains were found to form different populations according to the source of isolation, suggesting that geography may explain the difference. Contrary, strains of *S. cerevisiae* from sorghum beers produced in Burkina Faso, Côte d’Ivoire and Ghana clustered closely together despite the country of isolation, which according to the authors might be linked to human migration and thereby distribution of yeasts for inoculation through back-slopping or as dried yeast harvested from previous brews (Tapsoba et al., 2015). Diversity between *S. cerevisiae* strains isolated from indigenous sub-Saharan African food and beverages has also been reported within properties such as tolerance to acidic stress (Halm et al., 2004; Kubo et al., 2014; Houngbédji et al., 2019), ability to bind aflatoxin B_1_ (Shetty et al., 2007), production of tissue degrading enzymes (Padonou et al., 2010), folate synthesis (Hjortmo et al., 2008a), and phytase activity (Greppi et al., 2015).

Very few studies have been carried out on strain diversity among non-conventional yeast species isolated from indigenous sub-Saharan African fermented food and beverages, though some exist on *P. kudriavzevii.* By pheno- and genotypic characterisation of strains isolated from fermented maize dough it was found that several strains of *P. kudriavzevii* were involved from the onset of the fermentation and remained dominant throughout the fermentation (Hayford and Jakobsen, 1999). Strain diversity among isolates have been studied in indigenous sub-Saharan African fermented foods as, e.g., gowé, mawè, ogi and tchoukoutou by use of (GTG)_5_ rep-PCR genomic fingerprint patterns analysis (Greppi et al., 2013a; Houngbédji et al., 2018). For all fermented food products, strain clusters of *P. kudriavzevii* were reported. In mawè, diversities among the strains were found to vary between different mawè doughs. Especially raw materials, processing method and production site had an influence on the strain composition. Moreover, some strains were reported only to be linked to a single production site (Houngbédji et al., 2018). Strain variations within *K. marxianus* from the sorghum beer, bili bili, were studied and 12 groups of *K. marxianus* strains were isolated from the souring step based on physiological characteristics and their PCR/RFLP-NTS2 profiles. Based on chromosome polymorphism analysis by PFGE the *K. marxianus* strains could be divided into three different karyotypes (Maoura et al., 2005). Strains of *S. cerevisiae*, *P. kudriavzevii*, *K. marxianus*, and *C. glabrata* isolated from the solid food mawè was influenced in a species and strain dependent manner upon exposure to the intrinsic stress factors of the fermented cereal doughs, mawè, i.e., low pH, ethanol, lactic acid and acetic acid (Houngbédji et al., 2019).

## Yeasts and Their Interactions With the Commensal Microbiota

### Microbial Interactions in Fermented Food and Beverages

Besides a high number of yeast species, several other groups of microorganisms take part in the spontaneous fermentation of indigenous sub-Saharan African fermented food and beverages. Not surprisingly, the predominant group of microorganisms is LAB, but also other groups of bacteria might occur (Tamang et al., 2016). The high heterogeneity in the composition of most raw materials for fermented food and beverages allows simultaneous occupation of multiple niches by specialized species or strains, e.g., through utilization of different carbon sources (Sieuwerts et al., 2008).

Microbial interactions are quite common in spontaneous fermentations and do often involve interactions between several groups of microorganisms, i.e., yeast-yeast, bacteria-bacteria, yeast-bacteria and yeast-mold. Several interactions will occur simultaneously resulting in an interrelationship between different species continuously shaping the microbial consortium, while the interactions then become significant for obtaining the desirable characteristics of these foods (Viljoen, 2006). Microbial interactions can be divided into five main classes, i.e., antagonism, competition, commensalism, mutualism and parasitism (Sieuwerts et al., 2008; Smid and Lacroix, 2013), and sometimes the interactions even depend on direct physical contact or signaling molecules (Nissen et al., 2003; Skandamis and Nychas, 2012; Johansen and Jespersen, 2017). In the following, some examples of interactions among yeasts and other groups of microorganisms in indigenous sub-Saharan African fermented food and beverages are given.

### Yeast Interactions With Lactic Acid Bacteria (LAB)

Yeasts and LAB often co-exist during spontaneous fermentations. While yeasts can grow in a relatively simple medium, LAB are more fastidious and require more nutrients as, e.g., amino acids and vitamins (Viljoen, 2006; Ponomarova et al., 2017). The interactions between yeasts and LAB can be both synergistic and antagonistic, but are most often of mutual benefit (Romano et al., 2006). Mutualistic interactions between yeasts and LAB have been described for ogi, a non/low-alcoholic cereal-based beverage. While the yeasts will benefit from a decrease in pH by the acidification facilitated by the activity of the LAB, significant higher growth of *Lactobacillus plantarum* has been proved when co-cultured with either *S. cerevisiae* or especially *P. kudriavzevii*, indicating that these yeast species provide growth factors for the LAB (Omemu et al., 2007), most likely amino acids (Ponomarova et al., 2017). Mutualistic interactions have likewise been reported between species of LAB and yeasts originating from indigenous sub-Saharan African fermented milk products. When co-culturing *Lactococcus lactis* subsp. *lactis* biovar *diacetylactis* with *K. marxianus* in milk, the viability of *L. lactis* subsp. *lactis* biovar *diacetylactis* was enhanced, while *K. marxianus* could benefit from galactose, arising from lactose degradation by *L. lactis* subsp. *lactis* biovar *diacetylactis* (Gadaga et al., 2001a). Likewise, *Lactobacillus paracasei* subsp. *paracasei* reached significantly higher final counts when co-cultured in milk with especially *K. marxianus*, *S. cerevisiae* or *Naumovozyma dairenensis* (f. *Saccharomyces dairenensis*) (Gadaga et al., 2001b).

### Eukaryotic Interactions

The ability of some yeast species to inhibit molds has been proven for many foods (van den Tempel and Jakobsen, 2000; Masoud et al., 2005; Andrade et al., 2014; Núñez et al., 2015). Despite the relevance for many indigenous sub-Saharan African food products, practically no publications exist on the antagonistic effect of yeasts against molds in these type of fermented products. However, it has been shown that initial high counts of *Penicillium*, *Aspergillus*, and *Fusarium* species were significantly reduced during the first 24 h of fermentation of maize dough for kenkey production (Jespersen et al., 1994). Recently, more complex interaction mechanisms involving cell-to-cell communication such as, e.g., quorum sensing (QS) have been described among yeast species (Johansen and Jespersen, 2017). QS could be an important factor in regulating yeast community structures in indigenous sub-Saharan African fermented food and beverages, though not reported yet. Another interesting antagonistic interaction occurring between yeast species is the production of killer toxins and antimicrobial peptides (AMPs) (Marquina et al., 2002). Even though no reports on killer toxin or AMP producing yeasts exist for indigenous sub-Saharan African alcoholic beverages, it is most likely that killer or AMP producing yeasts could be present and be a determinant for the composition of the yeast consortium, especially when other microbial stress factors such as ethanol and organic acids are limited.

## Importance of Yeasts for Flavor

### Yeasts as Important Contributors to Flavor Formation

Indigenous sub-Saharan African fermented food and beverages are well appreciated for their natural tastes and flavors (Holzapfel, 2002). The microorganisms involved during the fermentation are able to produce a wide variety of compounds, which give to fermented foods their typical sensorial attributes. Formation of flavor compounds by conversion of carbohydrates is one of the most studied functions of yeasts, especially in the brewing industry (Jespersen, 2003). The main flavor compounds produced by yeasts during fermentation include alcohols, esters, organic acids, aldehydes and sulfur compounds (Dzialo et al., 2017). Several factors including the yeast species, intra-species variations and culture conditions affect the concentrations of these compounds being produced during the fermentation, and hence the flavor of the final product (Nedović et al., 2015). The importance of yeasts for the flavor formation in indigenous sub-Saharan African fermented food and beverages has, however, not been investigated to the same extent as is seen for much more industrialized fermented products as, e.g., ale and lager beers. A few examples on how yeasts influence the flavor formation in different types of indigenous sub-Saharan African fermented food and beverages are given below and in Table 5.

**TABLE 5:** Examples on the most common flavor compounds produced by yeasts in indigenous sub-Saharan African fermented food and beverages.

**Product type**	**Food name**	**Yeast(s)**	**Flavor compounds, specifically reported for the yeast species**	**References**
Solid food(cereal-based)	fermented maize dough	*S. cerevisiae*	Ethanol, propanol, 2-methylpropan-1-ol, 3-methylbutan-1-ol, ethyl acetate, ethyl-2-hydroxypropanoate, ethyl dodecanoate	Annan et al., 2003
		*P. kudriavzevii*	Acetic acid, 2-phenylethanol, hexyl acetate	
Non/low-alcoholic beverage (cereal-based)	obushera	*S. cerevisiae*	Ethanol, acetaldehyde, 2-methyl-1-propanal, 3-methyl-1-butanal, 2-methylbutan-1-ol, 3-methylbutan-1-ol	Mukisa et al., 2017
		*P. kudriavzevii*	Acetaldehyde, 2-methylpropan-1-ol, 2-methylbutan-1-ol, 3-methylbutan-1-ol	
Alcoholic beverage (cereal-based)	pito	*S. cerevisiae*	Ethanol, 2-methylpropan-1-ol, 3-methylbutan-1-ol	Demuyakor and Ohta, 1993
		Crude mixed yeast culture (from pito)	Acetaldehyde, ethyl acetate	
Alcoholic beverage (cereal-based)	ikigage	*S. cerevisiae*	Acetic acid, propanol, ethyl acetate, 2-methylpropan-1-ol, 3-methylbutan-1-ol	Lyumugabe et al., 2014
		*P. kudriavzevii*	Ethyl butanoate, ethyl octanoate, ethyl nonanoate, 2-methylpropyl butanoate, 2-phenylethanol	
Alcoholic beverage (fruit-based)	fermented masau	*S. cerevisiae*	Succinic acid, ethanol, propanol, 3-methylbutan-1-ol, ethyl hexanoate, ethyl octanoate	Nyanga et al., 2013
		*P. kudriavzevii*	Malic acid, succinic acid, ethyl acetate, ethyl octanoate	
		*C. fabianii*	Malic acid, succinic acid, 3-methyl-1-ol, 3-methylbutyl acetate, ethyl acetate, butyl acetate	
		*S. fibuligera*	Malic acid, succinic acid, 3-methyl-1-ol, 3-methylbutyl acetate, ethyl acetate, butyl acetate	
Alcoholic beverage (palm sap-based)	palm wine	*S. cerevisiae*	Acetaldehyde, propanol, 3-methylbutan-1-ol, 2-methylpropan-1-ol, ethyl acetate, ethyl butanoate	Hyma et al., 2011
Fermented milk	amasi	Spontaneous Back-slopping including yeasts	Citric acid, succinic acid, acetaldehyde, ethanol, 3-methylbutanal Lactic acid, succinic acid, ethanol	Gran et al., 2003

### Yeast as Specific Producers of Flavor Compounds

For solid foods, the flavor profile of fermented maize doughs produced through spontaneous fermentation or by use of starter cultures of *S. cerevisiae* and *P. kudriavzevii*, were significantly affected by the microorganisms involved in the fermentation (Annan et al., 2003). At the end of the fermentation, i.e., 72 h, 64 flavor compounds were identified in the doughs including 20 alcohols, 22 carbonyls, 11 esters, seven acids, one furan and three phenolic compounds, of which 51 were volatile. In the spontaneous fermentation, higher levels of carbonyl compounds were reported. In fermentations with *S. cerevisiae* as starter culture higher amounts of esters and fusel alcohols were reported, whereas *P. kudriavzevii* as starter culture resulted in higher levels of acetic acid and lower levels of most volatiles (Annan et al., 2003).

For non/low-alcoholic beverages, flavor compounds have been studied in the cereal-based togwa and obushera (Mugula et al., 2003a). The volatile flavor compounds detected in togwa based on sorghum or maize included ethanol, acetaldehyde, diacetyl, acetoin, 2-methylpropanal, 2-methylbutanal, 3-methylbutanal, 2-methylpropan-1-ol, 2-methylbutan-1-ol and 3-methylbutan-1-ol, with ethanol being the predominant volatile compound. Starter cultures for togwa fermentation comprising either LAB or co-cultures of LAB and *P. kudriavzevii* were tested for their fermentation properties and flavor development. Especially the co-cultures of LAB and *P. kudriavzevii* were reported to enhance the production of malty volatile flavor compounds, which are descriptive for togwa flavor (Mugula et al., 2003a). Obushera beverages fermented with different starter cultures comprising LAB or co-cultures of LAB and *S. cerevisiae* were evaluated for their flavor profiles. The co-culture of LAB and *S. cerevisiae* resulted in reduced amounts of lactate and diacetyl, and higher amounts of acetaldehyde and malty compounds, resulting in a flavor profile close to that of spontaneously fermented obushera. Further, only single cultures of *S. cerevisiae* and *P. kudriavzevii* were able to produce 2-methyl-propan-1-ol, 2-methyl-butan-1-ol and 3-methyl-butan-1-ol in significant amounts (Mukisa et al., 2017).

Flavor compounds in indigenous sub-Saharan African fermented products have been studied in several alcoholic beverages. In the sorghum beer pito, flavor compounds reported included alcohols, esters, and ketones in varying amounts depending on whether a single *S. cerevisiae* strain or crude mixed yeast cultures were used (Demuyakor and Ohta, 1993). The mixed yeast culture was reported to give a flavor typical of pito, while the single culture of *S. cerevisiae* produced an atypical pito with a dry and slightly bitter taste (Demuyakor and Ohta, 1993). In the sorghum beer ikigage, 55 flavor compounds belonging to esters, alcohols, acids and carbonyl groups were detected, which could be linked to the microorganisms used (Lyumugabe et al., 2014). A mixed culture, including *S. cerevisiae*, *P. kudriavzevii* and *Lactobacillus fermentum* were reported to produce ikigage beer with flavor and mouth feel similar to spontaneously fermented ikigage characterized by high concentrations of certain alcohols (propanol, 2-methylpropan-1-ol and butane-2,3-diol), esters (ethyl acetate, 2-methylpropyl acetate, propyl acetate, ethyl 2-hydroxypropanoate and ethyl pentanoate), organic acids (acetic acid and heptanoic acid) and carbonyls (acetaldehyde). The majority of these compounds were produced by *S. cerevisiae* and/or *P. kudriavzevii* (Lyumugabe et al., 2014). In the alcoholic beverage made from masau (*Ziziphus mauritania*) fruits the best ethanol producing *S. cerevisiae* strains, and strains of the species *P. kudriavzevii*, *Cyberlindnera fabianii* (anamorph *Candida fabianii*; f. *Pichia fabianii*), and *Saccharomycopsis fibuligera* were tested for production of flavor compounds (Nyanga et al., 2013). The major volatiles detected were alcohols and esters along with trace amounts of organic acids and carbonyl compounds. The yeast species produced significantly different amounts of ethanol and other volatile compounds. The highest amounts of ethanol and ethyl esters were reported for *S. cerevisiae*, though strains of *P. kudriavzevii*, *C. fabianii*, and *S. fibuligera* produced the highest amounts of ethyl acetate. Likewise, Hyma et al. (2011) reported that *S. cerevisiae* strains originating from palm wine in West Africa resulted in a different flavor formation in fermented grape musts than *S. cerevisiae* strains from vineyards or laboratory strains. The palm wine strain did especially produce acetaldehyde, propanol, 3-methylbutan-1-ol, 2-methylpropan-1-ol, ethyl acetate, and ethyl butanoate (Hyma et al., 2011).

In the fermented milk product amasi, flavor compounds were detected for spontaneously fermented milk, milk fermented using back-slopping and milk fermented with starter cultures of LAB (Gran et al., 2003). Spontaneously fermented milk and milk fermented with back-slopping, containing LAB and yeasts, were reported to have higher amounts of succinate, ethanol, malty aldehydes and methyl alcohols than the fermentations using pure starter cultures of LAB. The high levels of malty compounds in the spontaneously fermented milk and in milk fermented using back-slopping were suggested to arise from yeasts, which thereby highly impact the flavor of amasi (Gran et al., 2003).

## Nutritional Value and Health Benefits of Yeasts

The impact of fermentation on nutritional value and health benefits of fermented foods has been the focus of several studies, particularly focusing on bioavailability of micronutrients and probiotic properties. Some examples on the impact of yeasts in indigenous sub-Saharan African fermented food and beverages are given in Table 6.

**TABLE 6:** Examples on health beneficial properties of yeasts isolated from indigenous sub-Saharan African fermented food and beverages.

**Quality factor**	**Yeast species**	**Product type**	**Food name**	**Impact**	**References**
Phytase activity	*P. kudriavzevii*	Solid food, alcoholic beverage (cereal-based)	kenkey, pito	Species and strain dependent extracellular phytase activity.	Nuobariene et al., 2011
	*S. cerevisiae*				
	*P. kudriavzevii*	Alcoholic beverage, non/low alcoholic beverage (cereal-based)	burukutu, ogi, kunun-zaki	Species dependent extracellular phytase activity.	Ogunremi et al., 2015
	*P. kluyveri*				
	*C. tropicalis*	Non/low-alcoholic beverage (cereal-based)	ogi, kunun-zaki		
	*P. kudriavzevii*	Non/low-alcoholic beverage (cereal-based)	togwa	Species and strain dependent extracellular phytase activity. *P. kudriavzevii* and *H. guilliermondii* degrading 85–95% of initial IP_6_. No extracellular phytase activity was reported for several other yeast species.	Hellström et al., 2012, 2010
	*W. anomalus*				
	*K. marxianus*				
	*H. guilliermondii*				
	*P. kudriavzevii*	Solid food, alcoholic beverage and non/low-alcoholic beverage (cereal-based)	mawè, gowé, ogi, tchoukoutou	Species and strain dependent phytic acid utilization. *P. kudriavzevii* utilized phytic acid to highest extent. For *P. kudriavzevii*, extracellular phytase activity correlated with expression of phytase encoding gene (*PHYPk*).	Greppi et al., 2015
	*S. cerevisiae*				
	*C. lusitaniae*				
	*K. marxianus*				
	*M. farinosa*				
	*C. glabrata*				
	*W. anomalus*				
	*H. guilliermondii*				
	*D. nepalensis*				
Folate content	*P. kudriavzevii*	Non/low-alcoholic beverage (cereal-based)	togwa	Species and strain dependent 5-CH_3_-H_4_folate increase, up to 23-fold by *C. glabrata. S. cerevisiae*, highest specific total folate content.	Hjortmo et al., 2008a
	*S. cerevisiae*				
	*K. marxianus*				
	*C. glabrata*				
	*P. kudriavzevii*	Solid food (cereal-based)	pearl millet gruel similar to ben-saalga	Strain dependent total folate production, predominantly intracellular folate. *P. kudriavzevii* together with *L. fermentum* (co-culture) significantly increased total folate content compared to *L. fermentum* single cultures.	Greppi et al., 2017
Probiotic property	*P. kudriavzevii*	Solid food (cereal-based)	fura	Species and strain dependent survival at low pH (2.5) and in bile salts (0.3%). For *P. kudriavzevii*, *K. marxianus*, *C. rugosa*, *T. asahii*, relative TEER increased for Caco-2 and decreased for IPEC-2 monolayers.	Pedersen et al., 2012
	*K. marxianus*				
	*C. rugosa*				
	*T. asahii*				
	*C. fabianii*				
	*P. norvegensis*				
	*P. kluyveri*	Alcoholic and non/low-alcoholic beverages (cereal-based)	burukutu, kunun-zaki, ogi	Species and strain dependent survival at low pH (2.0–3.0), 37°C and in bile salts (0.3–2.0%). *P. kudriavzevii* and *P. kluyveri* had strong adhesive capabilities and co-aggregation with *E. coli*, *S. flexneri*, *S. paratyphi*. *P. kudriavzevii* and *P. kluyveri* showed *in vitro* reduction of cholesterol and antioxidant activity.	Ogunremi et al., 2015
	*P. kudriavzevii*				
	*P. kudriavzevii*	Solid food, alcoholic beverage and non/low-	mawè, gowé, ogi, tchoukoutou	Species and strain dependent survival at low pH (2.0) and in bile salts (0.3%) with *P. kudriavzevii* exhibiting strong survival. *P. kudriavzevii* exhibited strain dependent survival in synthetic gastric and pancreatic juices and strong adhesion capacity to Caco-2 monolayer.	Greppi et al., 2017
	*S. cerevisiae*				
	*C. lusitaniae*	alcoholic beverage (cereal-based)			
	*K. marxianus*				
	*M. farinosa*				
	*C. glabrata*				
	*W. anomalus*				
	*H. guilliermondii*				
	*D. nepalensis*				
	*S. cerevisiae*	Solid food, alcoholic beverage and fermented milk	kenkey, sorghum beer, suusac	Strain dependent adhesion to IPEC-J2 cells and pronounced reduction of IL-1α expression upon co-inoculation with *S. cerevisiae* strain A18 and *E. coli*.	van der Aa Kühle et al., 2005
Aflatoxin binding	*S. cerevisiae*	Solid food and alcoholic beverage (cereal-based)	kenkey, pito	Strain, growth phase and concentration dependent aflatoxin B_1_ surface binding.	Shetty et al., 2007
Degradation of hydrogen cyanide	*S. cerevisiae*	Solid food (tuber-based)	fermented cassava	Efficient HCN reduction by *S. cerevisiae* (65.9%).	Lambri et al., 2013

### Yeasts Affecting the Nutritional Value

In cereal-based foods, the presence of anti-nutritional factors such as the highly charged phytate limits the bioavailability of divalent ions through the process of chelation of cation minerals such as Fe^2+^, Zn^2+^, Ca^2+^, and Mg^2+^. The phytate complexes formed by chelation are insoluble at physiological pH and therefore, the divalent ions cannot be absorbed in the human intestine (Jespersen, 2003; Greppi et al., 2015). Microbial phytase activity has been reported for yeasts, particularly for *P. kudriavzevii.* Isolated from the solid fermented product kenkey (maize dough) and the alcoholic beverage pito (sorghum beer), strains of *P. kudriavzevii* and *S. cerevisiae* were reported to vary significantly in their extracellular phytase activity (Nuobariene et al., 2011). Similarly, strains of *C. tropicalis*, *Pichia kluyveri* and *P. kudriavzevii* isolated from burukutu (cereal beer), ogi and kunun-zaki (non/low-alcoholic beverages) were found to have high extracellular phytase activity (Ogunremi et al., 2015). Moreover, yeasts strains isolated from togwa, a non/low-alcoholic cereal-based fermented beverage were found to have significant phytase activities, with one strain of each of *H. guilliermondii* and *P. kudriavzevii* showing strong phytate degradation (≥95%) in a togwa model system (Hellström et al., 2010, 2012). Especially one strain of *P. kudriavzevii* (TY13) from togwa was shown to release substantial amounts of phytase enzyme to the environment. The extracellular phytase activity was found to be growth phase dependent, being highest in young growing cultures (Hellström et al., 2015). Phytase activity has also been reported for several different yeast species isolated from the solid foods mawè and gowé, the non/low-alcoholic beverage ogi and the alcoholic beverage tchoukoutou, with the highest phytase activity in *P. kudriavzevii* (Greppi et al., 2015). In the draft genome of *P. kudriavzevii* (M12) three phytase encoding genes were identified (Chan et al., 2012) and in Greppi et al. (2015), a correlation between the expression of these genes and extracellular phytase activity was established.

The production of folate (vitamin B_9_) by indigenous yeasts via fermentation is one way by which the nutritional value of cereal-based products can be improved. Unlike yeasts, the human body lacks the ability to synthesize folate and must therefore rely on sufficient intake from the diet (Hjortmo et al., 2008a). Yeasts isolated from indigenous sub-Saharan African fermented products have been evaluated for their capacity to produce folate in different studies. The production of folate by five yeast species originating from the non/low-alcoholic beverage togwa, i.e., *C. glabrata*, *K. marxianus P. kudriavzevii*, *S. cerevisiae*, and *W. anomalus* was determined in a model system of togwa by Hjortmo et al. (2008a). Folate production during fermentation were reported to vary depending on the yeast species and strain. Further, togwa fermented with yeasts contained 4 to 5-fold more folate than the un-inoculated control. Especially one *S. cerevisiae* strain (TY08) were shown to exhibit more than a 10-fold higher specific total folate content compared to other yeast strains (Hjortmo et al., 2008a). Besides species and strain dependent folate production, Hjortmo et al. (2008b) has shown that the specific folate content for *S. cerevisiae* vary extensively depending on cultivation medium, growth rate and growth state. Hence, controlling processing conditions could lead to significantly increased folate contents in the fermented food. In a food model mimicking the cereal-based gruel ben-saalga, total folate concentration was determined for co-cultures of *L. fermentum* and *P. kudriavzevii* as well as for LAB cultures alone. Here, fermentation of pearl millet with co-cultures of yeasts and LAB or LAB cultures alone were reported to increase the total folate concentration compared to unfermented pearl millet gruel. Furthermore, pearl millet gruels fermented with co-cultures of *P. kudriavzevii* and *L. fermentum* had significantly higher folate concentrations as compared to gruels fermented with LAB alone (Greppi et al., 2017).

### Probiotic Properties of Yeasts Isolated From Indigenous Sub-Saharan African Fermented Food and Beverages

While a wide diversity of yeasts have been isolated from indigenous sub-Saharan African fermented foods, specific yeast strains with proven probiotic properties are sparsely reported. However, few studies have reported the potential probiotic properties of yeasts isolated from sub-Saharan African fermented foods (van der Aa Kühle et al., 2005; Pedersen et al., 2012; Ogunremi et al., 2015; Greppi et al., 2017). The properties of seven yeast species from the solid food fura, based on fermented millet, were investigated by Pedersen et al. (2012). All the examined yeast species could survive at conditions equivalent to the human gastro-intestinal tract (GIT), i.e., at low pH (2.5), and in 0.3% (w/v) bile salts at 37°C. Furthermore, strains of *C. rugosa*, *K. marxianus*, *P. kudriavzevii* and *Trichosporon asahii* were reported to be able to increase the relative *trans*-epithelial electrical resistance (TEER) of polarized monolayers of human intestinal epithelial cells (Caco-2) in a manner comparable to the approved human probiotic yeast *S. cerevisiae* var. *boulardii* (Pedersen et al., 2012). In a similar study of the probiotic properties of one *P. kluyveri*, three *P. kudriavzevii* and one *C. tropicalis* strains isolated from cereal-based alcoholic beverage burukutu, and the non/low-alcoholic beverages kunun-zaki and ogi, all yeast strains survived at low pH (2.0), and 0.3–2.0% (w/v) bile salts at 37°C, with strain-specific variabilities (Ogunremi et al., 2015). Yeasts isolated from burukutu, kunun-zaki and ogi were able to co-aggregate with pathogenic bacteria, with the highest co-aggregation reported for *P. kudriavzevii* and *Escherichia coli* (Ogunremi et al., 2015). Yeasts isolated from different fermented cereal products including the solid foods gowé and mawè, the non/low-alcoholic ogi, and the alcoholic beverage tchoukoutou were likewise shown to be able to survive low pH (2.0), and 0.3% (w/v) of bile salts at 37°C, with isolates of *P. kudriavzevii* having the best survival rate in synthetic gastric and pancreatic juices (Greppi et al., 2017). Similar results were reported for *S. cerevisiae* strains isolated from different indigenous sub-Saharan African fermented foods, including the solid food kenkey, the alcoholic beverages pito and dolo, and fermented dairy product suusac (van der Aa Kühle et al., 2005). Two of the *S. cerevisiae* strains were additionally shown to be able to adhere to the intestinal porcine epithelial cell line IPEC-J2. Co-inoculation of IPEC-J2 cells with the adhesive *S. cerevisiae* strain A18 from sorghum beer, were reported to lead to pronounced reduction of IL-1α expression upon infection with *E. coli*. This indicated that *S. cerevisiae* A18 were able to lower the pro-inflammatory cytokine IL-1α response in a manner similar to what was observed for the human probiotic yeast *S. cerevisiae* var. *boulardii* (van der Aa Kühle et al., 2005).

### Reduction of Toxic Compounds by Yeasts Isolated From Indigenous Sub-Saharan African Fermented Foods

A strategy for reducing the deleterious effects of mycotoxins is biological decontamination by microorganisms, particularly by fermentation with LAB and yeasts. However, the ability of yeast isolates from indigenous sub-Saharan African fermented food to decontaminate or degrade mycotoxins is still sparsely investigated. Shetty et al. (2007) investigated the *in vitro* aflatoxin B_1_ binding abilities of 18 *S. cerevisiae* strains isolated from the solid food kenkey and the alcoholic beverage pito and reported that some strains could adsorb more than 40% of the added aflatoxin B_1_, and that the adsorption was strain specific but not dependent on the viability of the yeast strains.

Another group of toxic compounds, inherent to cassava tubers, are the cyanogenic glucosides linamarin and to a lesser extent lotaustralin, which have fatal consequences when consumed in unprocessed cassava. Fermentation has been widely documented as a way to enhance the safety, organoleptic and nutritional quality of many cassava-derived foods (Holzapfel, 2002), and *S. cerevisiae* has additionally been demonstrated to reduce the total cyanide content, e.g., in fermented cassava tubers from Burundi (Lambri et al., 2013).

## Safety; Opportunistic Pathogenic Yeasts

As indigenous sub-Saharan African food and beverages predominantly are produced by spontaneous fermentation, the consumers may be exposed to large populations of different yeast species of often unknown origin (Ogunremi et al., 2017). Fortunately, unlike other microbial groups, food borne yeasts are generally not considered to be aggressive pathogens and are rarely associated with outbreaks of foodborne gastroenteritis, intoxications or other infections (Fleet, 2007; Ogunremi et al., 2017). Nonetheless, caution is needed particularly in selecting yeast strains for the development of starter cultures, as some species are capable of causing disease in especially immunocompromised individuals. Many species of the genus *Candida* are widely distributed in nature and have frequently been isolated from indigenous sub-Saharan African fermented products. Most of them are harmless, but some *Candida* spp. can take advantage of a locally or systemically impaired immune system to proliferate in the host and cause diseases generally termed “candidiasis” (Papon et al., 2013). *C. albicans* is the most frequently isolated agent of candidiasis, causing about 41–47% of total yeast infections worldwide (Pfaller and Castanheira, 2016). However, non-*albicans* candidiasis infections are also widespread with worldwide prevalence in invasive candidiasis as follows: *C*. *glabrata* (18–27%), *C*. *parapsilosis* (16–18%), *C*. *tropicalis* (9–11%), and *C*. *krusei* (1–3%) (Pfaller and Castanheira, 2016).

Generally, *C. albicans* is only found in few indigenous sub-Saharan African fermented foods, occurring in low numbers in the fermented milk products amabere amaruranu and sethemi (Kebede et al., 2007; Nyambane et al., 2014) as well as in the sorghum beer tchoukoutou (Kayode et al., 2011). *C. glabrata* has been reported in some indigenous sub-Saharan African fermented food and beverages, including gari, lafun, mawè, tchoukoutou, and togwa (Tables 1–3). Both yeasts are most probably occurring as a result of contamination during processing due to improper human handling. Contrary, *C. tropicalis* is frequently identified in indigenous sub-Saharan African fermented food and beverages, including akyeke, amasi, attiéké, bandji, fufu, fura, gari, gowé, kaffir, lafun, mawè, mukumbi, nunu, palm wine, pito, sethemi, tchapalo and teff-injera (Tables 1–4). *Candida* spp. have emerged as major agents of human mucosal, systemic and bloodstream yeast infections (Silva et al., 2012; Pfaller and Castanheira, 2016). Moreover, examples of resistance to well-known antifungal drugs have been reported, e.g., *C*. *glabrata* is particularly prone to develop resistance to fluconazole, a first-line antifungal treatment for yeast infections (Abbes et al., 2011), and resistance to fluconazole and other azoles appears to be increasing among clinical isolates of *C. tropicalis* (Pfaller and Castanheira, 2016). Consequently, further research is needed to optimize fermentation conditions to eliminate opportunistic pathogenic yeasts during processing and equally important, improved hygienic conditions need to be ensured in order to prevent cross-contamination from human handling during food processing.

As apparent from the above, *P. kudriavzevii* under different names, i.e., *C. krusei* and *I. orientalis* (Kudryavtsev, 1960; Kurtzman et al., 2008) is very often encountered in indigenous sub-Saharan African fermented food and beverages and plays key technological functions i.e., phytase production, flavor formation, etc. (Oyewole, 2001; Jespersen, 2003; Oguntoyinbo, 2008; Yadav et al., 2012; Greppi et al., 2015). It has recently been confirmed that *C. krusei* and *P. kudriavzevii* are the same species, and that there is no genetic distinction between clinical isolates of *C. krusei* and environmental isolates of *P. kudriavzevii* (Douglass et al., 2018). Traditionally, *P. kudriavzevii* has been considered as non-pathogenic due to its occurrence and technological role in many fermented food products. However, the species draws attention regarding the safety of its use in food processing due to its opportunistic pathogenic traits. Additionally, *C. krusei* (*P. kudriavzevii*) has been found to have innate resistance to widely used triazole antifungal drugs, particularly fluconazole, and has in some cases been reported to lead to mortality in immunocompromised individuals (Lamping et al., 2009; Garnacho-Montero et al., 2010). Consequently, *C. krusei* is not considered “generally regarded as safe” by the US Food and Drugs Administration nor included in the quality presumptive safety (QPS) list of the European Food Safety Authority (EFSA). Because of the taxonomic identity between *P. kudriavzevii* and *C. krusei*, the proven close relatedness with clinical isolates of *C. krusei* and the high resistance to fluconazole by the species, some strains of *P. kudriavzevii* could present potential health hazards to immunocompromised consumers (Douglass et al., 2018). As no studies have been carried out on the variability in potential pathogenic traits among strains of *P. kudriavzevii* isolated from indigenous sub-Saharan African food and beverages this topic still calls for investigation.

## Future Perspectives for Up-Grading Indigenous Sub-Saharan African Fermented Food and Beverages

The market size of indigenous sub-Saharan African fermented food and beverages are growing, among others due to their ability to be used as convenient food by consumers. Additionally, fermentation is an affordable and sustainable way of processing that easily can be used to improve the quality and safety of food and beverages. Unfortunately, uncontrolled processing, handling and selling on streets may induce contamination and growth of harmful microorganisms. The challenges will be to develop better starter cultures targeted indigenous sub-Saharan African fermented food and beverages and to optimize the processing. To achieve this, several studies have been carried out to study the biochemical and microbiological changes which occur during processing including information on nutritional value and consumer preferences (Halm et al., 1996; Mugula et al., 2003b; Edema and Sanni, 2008; Padonou et al., 2010; Greffeuille et al., 2011; Akabanda et al., 2013; Houngbédji et al., 2018). Some of these studies have demonstrated that when the yeasts are combined in consortia it makes the fermented products more appealing. However, there is still a need to understand the organization of the consortium, the species-species interactions and species-environment interactions. In parallel, the need to select and develop starter cultures of non-pathogenic strains of yeasts with permissible levels of drug resistance for application in indigenous sub-Saharan African fermented food products is obvious.

To generate sufficient data for large-scale production in SMEs, optimal processing parameters such as raw material preparation as well as ways of microbial propagation and inoculation should be investigated further. Achievement of these challenges will help to develop these indigenous products as novel foods for an international market. The knowledge generated should be used to optimize processing in order to control the microbial community during fermentation for optimized quality, shelf life, safety, nutritional value and other health benefits. The success of indigenous sub-Saharan African fermented food and beverages is also based on the collection of appropriate information concerning consumers’ needs and expectations that are essential tools for building competitive advantage and long-term SMEs success in the market and for prevention of negative changes in product quality and acceptability, consumers’ complaints and product rejection.

## Conclusion and Perspectives

The present survey has proved that a significant number of yeast species are involved in the fermentation of indigenous sub-Saharan African food and beverages. Despite the fact that these fermented products play a significant role in the diet of sub-Saharan African inhabitants, they are not investigated to the same extent as their industrialized counterparts. Consequently, the potential of these products is not explored and even worse, their sustainability is under pressure. From a taxonomic point of view, it is clear that many of the yeast species found in these spontaneously fermented products could add significantly to our understanding of the evolution of yeast. Likewise, it becomes clear that these yeasts, to a greater extent, should be described in current taxonomic keys. Studying spontaneous fermentations offers a valuable scenario for understanding microbial interactions at various levels. Cross-kingdom interactions occur when yeasts and LAB evolve together. Yeasts acts as bio-controlling agents preventing the growth of pathogenic bacteria and mycotoxin producing molds, and at the intra-species level, an often overlooked battle is going on. As proved in the current review, strains within the same species often have different traits evolved as a consequence of their adaption to specific ecological niches. Strain variations are not only seen for industrialized processes such as in the production of barley beer and wine but similar strain variations exist within the yeast species involved in spontaneous fermentation of food and beverages in sub-Saharan Africa. This point toward an unexplored microbial heritage which could offer, not only valuable academic insight, but new enzymes, cell wall components, killer toxins, etc., with new interesting properties relevant for the biotechnological and biomedical sectors.

The predominant yeast species involved in the fermentation of indigenous sub-Saharan African fermented food and beverages are mostly harmless and do hardly ever cause any infections, contrary they are able to produce essential compounds as folate, enhance bioavailability by phytate degradation, degrade toxic compounds as linamarin, preventing uptake of toxins as, e.g., aflatoxin B_1_ in the human GIT, provide probiotic properties, etc. However, some species have been reported as having opportunistic pathogenic traits. This is especially of relevance for *P. kudriavzevii*, which according to the present survey, together with *S. cerevisiae* is the far most predominant yeast species in indigenous fermented sub-Saharan African food and beverages. As a paradox, *P. kudriavzevii* has also been proved, among several other yeast species, to be high in phytase and folate production, thereby offering a substantial improvement of spontaneously fermented foods in sub-Saharan Africa. The present survey calls for solid research to be conducted on strain variations in properties related to product quality and health concerns. *C. glabrata*, generally recognized as a human pathogen, does occasionally occur in indigenous fermented sub-Saharan African foods most likely due to unhygienic handling procedures. However, only limited knowledge exists on how to prevent the growth of this species during fermentation.

In conclusion, yeasts make a significant contribution to the fermentation of many food and beverages produced in sub-Saharan Africa. However, academic knowledge is still required, strain variations are still unexplored, methods for production of starter cultures need to be upgraded and methodologies for in-depth understanding of microbial interactions need to be developed. Only by going in-depth within these topics we can ensure the sustainability of these indigenous fermented products being very important not only for in-come generation but also for the nutrition of many people in sub-Saharan Africa.

## Author Contributions

LJ designed the manuscript. All authors wrote the manuscript. PJ and LJ critically revised the manuscript.

## Conflict of Interest Statement

The authors declare that the research was conducted in the absence of any commercial or financial relationships that could be construed as a potential conflict of interest.
